# Molecular Tracing to Find Source of Protracted Invasive Listeriosis Outbreak, Southern Germany, 2012–2016

**DOI:** 10.3201/eid2310.161623

**Published:** 2017-10

**Authors:** Sylvia Kleta, Jens Andre Hammerl, Ralf Dieckmann, Burkhard Malorny, Maria Borowiak, Sven Halbedel, Rita Prager, Eva Trost, Antje Flieger, Hendrik Wilking, Sabine Vygen-Bonnet, Ulrich Busch, Ute Messelhäußer, Sabine Horlacher, Katharina Schönberger, Dorothee Lohr, Elisabeth Aichinger, Petra Luber, Andreas Hensel, Sascha Al Dahouk

**Affiliations:** German Federal Institute for Risk Assessment, Berlin, Germany (S. Kleta, J.A. Hammerl, R. Dieckmann, B. Malorny, M. Borowiak, A. Hensel, S. Al Dahouk);; Robert Koch Institute, Berlin (H. Wilking, S. Vygen-Bonnet);; Robert Koch Institute, Wernigerode, Germany (S. Halbedel, R. Prager, E. Trost, A. Flieger);; Bavarian Health and Food Safety Authority, Oberschleißheim, Germany (U. Busch, U. Messelhäußer, K. Schönberger);; Chemical and Veterinary Investigatory Office, Stuttgart, Germany (S. Horlacher);; Baden-Württemberg State Health Office, Stuttgart (D. Lohr, E. Aichinger);; Federal Office of Consumer Protection and Food Safety, Berlin (P. Luber)

**Keywords:** *Listeria monocytogenes*, bacteria, listeriosis, foodborne disease, outbreak, Germany, whole-genome sequencing, molecular tracing, pulsed-field gel electrophoresis, molecular typing, forensic microbiology, food safety

## Abstract

We investigated 543 *Listeria monocytogenes* isolates from food having a temporal and spatial distribution compatible with that of the invasive listeriosis outbreak occurring 2012–2016 in southern Germany. Using forensic microbiology, we identified several products from 1 manufacturer contaminated with the outbreak genotype. Continuous molecular surveillance of food isolates could prevent such outbreaks.

Listeriosis is a serious, life-threatening infectious disease caused by *Listeria* *monocytogenes* that mainly affects elderly and immunocompromised persons, pregnant women, and neonates (*1**,**2*). In Germany, listeriosis cases have been predominantly reported among nonpregnant women and men, and the source of most infections was unknown (*3*). The major risk factors identified for these cases were immunosuppressive therapy, immunocompromising diseases, gastric acid suppression, and frequently consumed ready-to-eat foods (i.e., packed and presliced cheese and boiled sausages) (*4*). *L. monocytogenes* is usually transmitted through food prone to contamination during manufacturing or postproduction processing before packing. In 2012, the number of *L. monocytogenes* cases in Germany started continuously increasing; 707 cases (incidence rate 0.9/100,000 population) and a case-fatality rate of 7% were reported in 2016 (*3*). Among the 6 most predominant enteric pathogens in Germany, *L. monocytogenes* has accounted for the highest number of years of potential life lost (*5*).

## The Study

At the end of 2015, an outbreak of invasive listeriosis caused by *L. monocytogenes* serotype 1/2a was reported in southern Germany (*6*). The outbreak became apparent because analysis of the *L. monocytogenes* isolates from patients residing in the federal states Bavaria, Baden-Württemberg, and Hesse revealed the same novel pulsed-field gel electrophoresis (PFGE) pattern: 13a/54. As of July 14, 2017, the National Reference Centre for *Salmonella* and Other Bacterial Enteric Pathogens and the Binational Consiliary Laboratory for *Listeria*, a collaborative agreement between the Robert Koch Institute (Wernigerode, Germany) and the Austrian Agency for Health and Food Safety (Wien, Austria), had received 84 human isolates with this PFGE pattern. These isolates could be assigned to 78 surveillance cases reported in the national mandatory notification system. Subsequent whole-genome sequencing (WGS) and core-genome multilocus sequence typing (MLST) (*7*) resulted in the assignment of 57 human isolates to the unique cluster type 1248 (sequence type 8 according to the Institut Pasteur MLST database; http://bigsdb.pasteur.fr/). A possible case was defined as signs and symptoms of acute invasive listeriosis in a patient with disease onset November 2012–October 2016 from which *L. monocytogenes* with the PFGE pattern 13a/54 was isolated. Confirmed cases met the aforementioned criteria and included assignment of the *L. monocytogenes* isolate into the core-genome MLST cluster type 1248 (*6*). Analysis of the patients’ food consumption habits, which were recorded during explorative interviews, did not identify the causative food item.

We analyzed 543 environmental *L. monocytogenes* isolates of molecular serotype IIa (comprising the conventional serotypes 1/2a and 3a) that corresponded with the spatial and temporal distribution of cases in southern Germany. Staff of official food control laboratories had acquired these isolates from food matrices and food processing plants in the affected federal states. With these isolates, we performed PFGE analysis (with only *Apa*I for initial screening) and WGS at the National Reference Laboratory for *L. monocytogenes* (Berlin, Germany).

**Figure 1:**
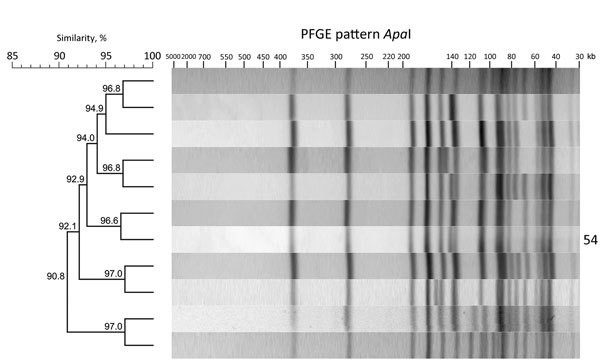
*Apa*I restriction enzyme analysis of *Listeria monocytogenes* outbreak strain and isolates with >90% similarity to the outbreak strain, southern Germany, 2012–2016. We performed molecular subtyping in line with the PulseNet standardized PFGE protocol for *L. monocytogenes* (*8*) and the standard operating procedures of the European Union Reference Laboratory for *L. monocytogenes* (*9*) to ensure interlaboratory comparability of the results. We analyzed PFGE patterns using BioNumerics software version 7.5 (Applied Maths, Sint-Martens-Latem, Belgium). Dendrogram indicates percentage similarity between the *Apa*I PFGE pattern of the outbreak strain and that of the other closely related isolates. Outbreak strain PFGE pattern is labeled with the number 54. PFGE, pulsed-field gel electrophoresis.

**Figure 2:**
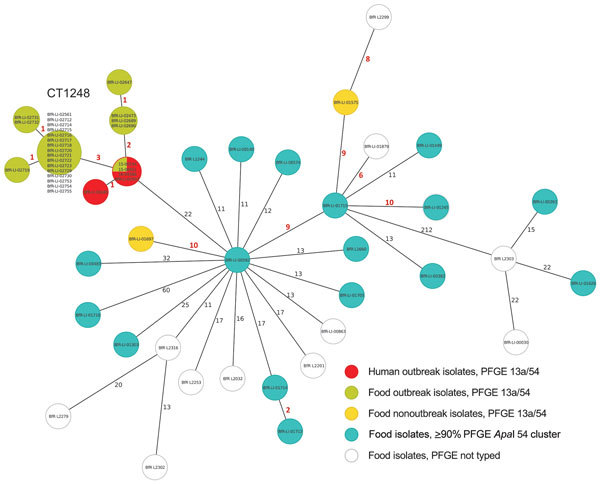
Minimum spanning tree estimating the phylogenetic relationships among outbreak and nonoutbreak *Listeria monocytogenes* isolates from humans and from food products, southern Germany, 2012–2016. We conducted bioinformatics analyses using the Ridom SeqSphere+ software version 3.1.0-2016-01 (Ridom GmbH, Münster, Germany). The core-genome multilocus sequence typing scheme for whole-genome sequencing–based typing of *L. monocytogenes* relies on a set of 1,701 target genes (alleles) that are present in >99% of the known genomes of the species (*7*). Each circle represents an allelic profile. The numbers on the connecting lines illustrate the number of differing alleles in a pairwise comparison. Closely related genotypes (<10 allele difference) designated a cluster type and are indicated with bold red numbers. CT, cluster type; PFGE, pulsed-field gel electrophoresis.

Analysis showed that 26 isolates from food had the same *Asc*I (13a) and *Apa*I (54) restriction patterns as the human outbreak strain; patterns from 55 isolates showed >90% similarity to the PFGE pattern *Apa*I 54 (Figure 1). We sequenced all 13a/54 isolates and 17 of the 55 isolates representative of the cluster with >90% similarity. In addition, 148 isolates were directly subjected to WGS. We conducted comparative genomic analyses to find single-nucleotide polymorphisms (Technical Appendix Figure) and compared the isolates closely related to the outbreak strain by core-genome MLST (Figure 2) (*8*). We defined the threshold for strain affiliation with the outbreak cluster as a difference of <10 alleles (*7*).

In May 2016, we identified an *L. monocytogenes* isolate (BfR-LI-02473) with the 13a/54 PFGE pattern that belonged to the core-genome MLST cluster type 1248. This isolate had been found at a retail store in Bavaria 2 months prior in a smoked pork belly sampled by food inspectors. The meat product had been manufactured by a meat processing plant in Germany that distributed food products in southern Germany. The highly contaminated batch of smoked pork belly (bacterial concentration 1.9 × 10^5^ CFU/g) was recalled from consumers and withdrawn from the market.

Follow-up investigations of this meat processing plant revealed isolates identical to BfR-LI-02473 (BfR-LI-02689, BfR-LI-02690, and BfR-LI-02647) in another batch of smoked pork belly, in vegetarian sausages, and in 2 types of boiled pork sausages. The bacterial concentration in these products was <100 CFU/g. At the end of May 2016, all food products from this meat producer were banned from sale, and those already on the market were withdrawn.

Two more core-genome MLST cluster types found in raw meat and sausage were identified in the company’s production chain. However, based on the PFGE data, we could not assign these isolates to any human cases that occurred in southern Germany during 2012–2016. Furthermore, an *L. monocytogenes* isolate obtained from the fecal sample of an employee differed from the outbreak strain. Environmental sampling of the suspected plant led to the identification of a potential contamination hotspot: a conveyor belt on which food products were directly placed before packaging. However, no isolates were available for sequencing to confirm this hypothesis.

Overall, this outbreak investigation revealed 26 isolates from food with the 13a/54 PFGE pattern: 24 isolates either originating from or associated with food products from the suspected outbreak source assigned to the cluster type 1248 and 2 isolates not epidemiologically linked to the suspected outbreak source that differed from cluster type 1248 by >32 alleles. Isolates closely related to the outbreak strain and included in cluster B (Technical Appendix Figure) differed by >22 alleles by core-genome MLST analysis (Figure 2). The human outbreak isolates differed from each other by <3 alleles, and among all human isolates, the maximum difference was 8 (*6*). Compared with the human outbreak isolates, the outbreak isolates from food having the 13a/54 PFGE pattern and belonging to the core-genome MLST cluster type 1248 contained only 2–4 allele differences based on single-nucleotide polymorphisms, indicating a close phylogenetic relationship (Figure 2). On-site investigation in the household of a patient who regularly consumed smoked pork belly distributed by the suspected outbreak source revealed an isolate in cheese belonging to cluster type 1248 and identical to the patient isolate. Comparative analyses of unopened packages of the same batch of cheese were negative for *L. monocytogenes*, suggesting cross-contamination in the patient’s household.

The meat processing plant predominantly supplied grocery stores of a single company. Patients and their relatives often shopped at these grocery stores and frequently ate pork products. Altogether, food consumption histories of patients were compatible with our molecular typing results, but we could not prove the producer was the source of the infections. After the production plant was shut down, the outbreak strain was isolated from only 3 more persons who either might have consumed or consumed pork products from this company with a high degree of probability.

## Conclusions

The isolation of the *L. monocytogenes* outbreak strain in various food products from the same manufacturer, absence of the outbreak strain in a large number of food products collected during the outbreak from the same region, and subsequent epidemiologic findings suggested that the source of outbreak had been identified. Epidemiologic analysis did not provide the information needed to determine the outbreak source; thus, forensic microbiology based on WGS of *L. monocytogenes* isolates from patients and food became essential to take the appropriate countermeasures. Public health could benefit from continuous molecular surveillance of isolates from humans and food, which could allow for infectious disease outbreaks to be stopped before emergence.

Technical Appendix Maximum parsimony tree of *Listeria monocytogenes* isolates collected in Bavaria, Baden-Württemberg, and Hesse, Germany, 2012–2016.
