# Supplementary material for: Molecular Tracing to Find Source of Protracted Invasive Listeriosis Outbreak, Southern Germany, 2012–2016
Source: Emerg Infect Dis. 2017 Oct;23(10):1680–3. doi: 10.3201/eid2310.161623 (PMC5621528; doi:10.3201/eid2310.161623)
Supplement: Technical Appendix — Maximum parsimony tree of Listeria monocytogenes isolates collected in Bavaria, Baden-Württemberg, and Hesse, Germany, 2012–2016. [file 16-1623-Techapp-s1.pdf]

Article DOI: https://doi.org/10.3201/eid2310.161623

Molecular Tracing to Find Source of Protracted Invasive Listeriosis Outbreak, Southern Germany, 2012–2016

Technical Appendix

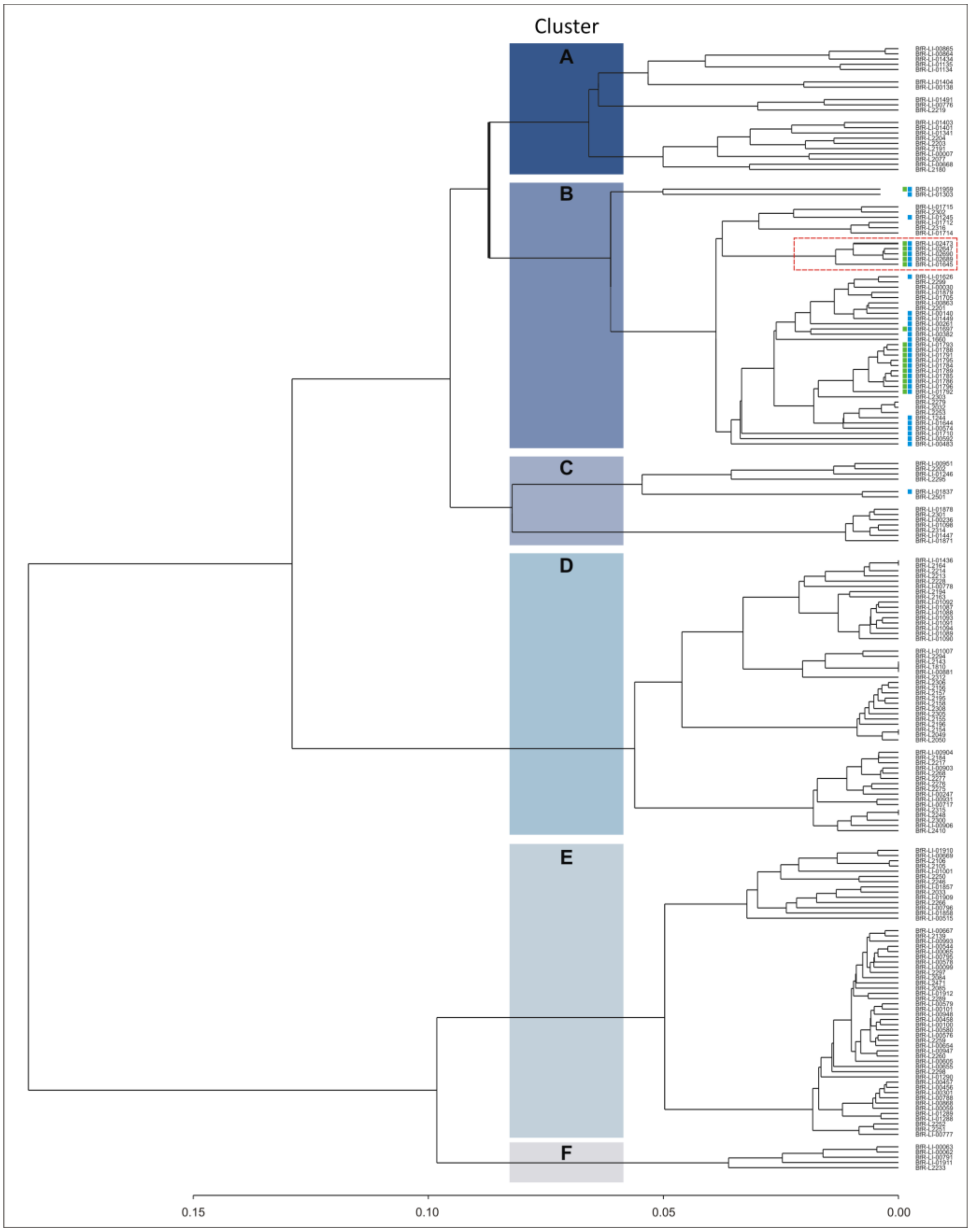

Technical Appendix Figure. Maximum parsimony tree of 174 *Listeria monocytogenes* isolates from food samples collected in Bavaria, Baden-Württemberg, and Hesse, Germany, 2012–2016. For mapping and single nucleotide polymorphism calling, we used an in-house pipeline of Eurofins Medigenomix (Ebersberg, Germany). The red box displays the outbreak cluster including a human isolate (BfR-LI-01645, PFGE 13a/54, core-genome MLST cluster type 1248) and isolates from smoked pork bellies that were distributed by the meat manufacturing plant suspected of being the outbreak source. Another 14 *L. monocytogenes* isolates from food that were not related to the outbreak but displayed the PFGE pattern 13a/54 (green squares) or PFGE pattern similar to ($\geq$90%) *Apa*I 54 (blue squares) were included in sequence analysis. Scale bar indicates mean number of nucleotide substitutions per site. MLST, multilocus sequence typing; PFGE, pulsed-field gel electrophoresis.
